# Accumulation of Extracellular Matrix in Advanced Lesions of Canine Distemper Demyelinating Encephalitis

**DOI:** 10.1371/journal.pone.0159752

**Published:** 2016-07-21

**Authors:** Frauke Seehusen, Seham A. Al-Azreg, Barbara B. Raddatz, Verena Haist, Christina Puff, Ingo Spitzbarth, Reiner Ulrich, Wolfgang Baumgärtner

**Affiliations:** 1 Department of Pathology, University of Veterinary Medicine, Hannover, Germany; 2 Boehringer Ingelheim Veterinary Research Center GmbH & Co. KG, Hannover, Germany; 3 Friedrich-Loeffler-Institut, Federal Research Institute for Animal Health, Greifswald - Insel Riems, Germany; University of Crete, GREECE

## Abstract

In demyelinating diseases, changes in the quality and quantity of the extracellular matrix (ECM) may contribute to demyelination and failure of myelin repair and axonal sprouting, especially in chronic lesions. To characterize changes in the ECM in canine distemper demyelinating leukoencephalitis (DL), histochemical and immunohistochemical investigations of formalin-fixed paraffin-embedded cerebella using azan, picrosirius red and Gomori`s silver stain as well as antibodies directed against aggrecan, type I and IV collagen, fibronectin, laminin and phosphacan showed alterations of the ECM in CDV-infected dogs. A significantly increased amount of aggrecan was detected in early and late white matter lesions. In addition, the positive signal for collagens I and IV as well as fibronectin was significantly increased in late lesions. Conversely, the expression of phosphacan was significantly decreased in early and more pronounced in late lesions compared to controls. Furthermore, a set of genes involved in ECM was extracted from a publically available microarray data set and was analyzed for differential gene expression. Gene expression of ECM molecules, their biosynthesis pathways, and pro-fibrotic factors was mildly up-regulated whereas expression of matrix remodeling enzymes was up-regulated to a relatively higher extent. Summarized, the observed findings indicate that changes in the quality and content of ECM molecules represent important, mainly post-transcriptional features in advanced canine distemper lesions. Considering the insufficiency of morphological regeneration in chronic distemper lesions, the accumulated ECM seems to play a crucial role upon regenerative processes and may explain the relatively small regenerative potential in late stages of this disease.

## Introduction

Canine distemper virus (CDV), a morbillivirus of the family *Paramyxoviridae*, commonly causes central nervous system (CNS) disease [1,2]. Though both gray and white matter infection and disease can be observed in distemper, demyelinating leukoencephalomyelitis (DL) represents the major manifestation of CDV-induced encephalitis in dogs [3]. In DL, initial alteration of the gray matter is followed by viral spread into the white matter [3]. With regard to the morphology of the lesions, DL shares some features with human demyelinating diseases such as Multiple Sclerosis (MS), which has consequently led to the use of DL as a spontaneously occurring animal model of demyelination [1,4,5]. Based on the age of the lesions in DL, distinct lesion types with specific morphological features are distinguished [3,6–9]. As a proposed biphasic process, the early phase of demyelination in DL is believed to be attributed to direct virus effects [10,11]. During early demyelination, there is an infiltration of CD8-positive cytotoxic T cells, paralleled by anup-regulation of proinflammatory cytokines such as interleukin (IL)-6, IL-8, tumor necrosis factor (TNF)-α and IL-12 [10,11]. Conversely, demyelination in advanced phases of the disease, which are characterized by decreasing viral protein expression, is supposed to be based on immunopathologic processes, as indicated by strong up-regulation of MHC class II, interferon-γ and IL-1 as well as immune response dominated by CD4- and CD8-positive lymphocytes, plasma cells and macrophages [1].

Collagens, fibronectin and laminin build the main mass of the total extracellular matrix (ECM) in most tissues while only small amounts of these molecules are found in the brain and spinal cord, where they form the basement membranes as a part of the blood-brain barrier (BBB). In contrast to these ubiquitous components, other molecules including brevican, neurocan, phosphacan and tenascin-R that are found exclusively in the central nervous system [12,13]. The expression of matrix molecules of the CNS is finely regulated during the pre- and postnatal phase and they are produced in a temporal-spatial pattern [14–19]. The main sources of ECM molecules in the CNS are neurons and glial cells. Additionally, endothelial cells play an important role for the production of basement membrane glycoproteins [12,20,21].

In canine distemper demyelinated plaques, the cell surface receptor for the ECM molecule hyaluronate (CD44) is mainly located on astrocytes and upregulated in acute and subacute demyelination [22]. There is a decreased immunoreactivity of CD44 in chronic plaques and additional expression on perivascular mononuclear cells [22]. Matrix-metalloproteinases (MMPs) are important zinc dependent enzymes that degrade the ECM and therefore can lead to breakdown of the blood-brain-barrier [23]. In distemper, they are most prominently upregulated in acute and subacute non-inflammatory lesions. In chronic lesions, expression of MMPs and their inhibitors (tissue inhibitors of metalloproteinases, TIMPs) decrease apart from MMP-11, -12, and -13. CD44 and MMPs might be associated with onset of demyelination and may initiate ECM disturbances [22].

Regenerative attempts are rare events in DL. In subacute lesions, there is an occurrence of p75^NTR^-positive cells, possibly representing a pre-myelinating stage of Schwann cells [24]. Nevertheless, only single periaxin-positive cells which are suggestive of manifest Schwann cell remyelination, were detected within few advanced lesions [25]. Furthermore, it was also shown that following extensive axonal degeneration the axonal expression of GAP43, indicative of axonal regeneration, failed to reach the level of significance in dogs with CDV-DL [25]. The up-regulation of ECM molecules, especially of chondroitin sulfate proteoglycans, after CNS injury is known to impair axonal sprouting and inhibit remyelination by oligodendroglial cells [26,27]. Nevertheless, the influence of ECM molecules upon regenerative effects in CDV-DL has not been investigated so far.

In this study, the spatiotemporal distribution of ECM molecules in the cerebella of CDV-infected dogs was investigated *in silico* by microarray analysis as well as in *post mortem* tissue by histochemistry and immunohistochemistry.

## Materials and Methods

### Ethics statement

All formalin-fixed and paraffin embedded archived and frozen material used in this study was collected by one of the authors (WB) during his work at the diagnostic pathology services of the Department of Pathology, University of Veterinary Medicine Hannover and Justus Liebig University, Gießen. The majority of the brain samples have been used in a previous study [28]. The present study was conducted in accordance with the German Animal Welfare Act. The authors confirm that no animals were infected or sacrificed for the purpose of this retrospective pathological study. This study is not an animal experiment since all animals were dead at the time of submission for necropsy in order to investigate the causes of death and disease. In cases in which euthanasia was performed because of poor prognosis, this procedure was done in the respective Veterinary Hospital before the patient was submitted to the diagnostic service of the Department of Pathology. All dog owners provided written consent for the dogs’ tissues to be collected and used for research purposes.

### Histochemical and immunhistochemical investigations

Serial sections of archived formalin-fixed, paraffin-embedded cerebella of 15 dogs suffering from CDV-DL and 4 healthy control animals (group 1) were processed as described [28]. Light microscopic changes in the cerebellum of the diseased dogs were subdivided into seven lesion groups and one control group as described (Table 1; [28]).

**Table 1:** Numbers of animals, investigated areas and groups in histochemistry and immunohistochemistry.

Group no.	1	2	3	4	5	6	7	8
Definition	control	Normal appearing white matter (NAWM), no CDV antigen, no lesion	Antigen without lesion, detection of CDV antigen	Vacuolation of WM, detection of CDV antigen	acute lesion	subacute non-inflammatory lesion, demyelination	subacute inflammatory lesion, demyelination	chronic lesion, demyelination and inflammation
Investigated areas	20	34	40	8	31	18	8	10
No. of animals	4	15	13	4	9	9	5	2

In the cerebellum of CDV-infected dogs, 2 to 3 areas from each animal which showed no lesions in the H&E staining and also no CDV-NP immunoreactivity were selected and were considered NAWM (group 2, n = 34). The lesioned areas were divided into group 3 to group 8 as described [28]. Sometimes all lesions types were detected in one section of one animal so that the final neuropathological diagnosis was based on the most advanced type of white matter lesions. Summarized, a total of 169 cerebellar areas were investigated (Table 1).

Various histochemical stains (azan stain for mucopolysaccharides; modified picrosirius red [PSR] stain for collagens and proteoglycans; Gomori´s silver stain for reticular and collagen fibers) [26,29] and immunohistochemistry with antibodies directed against different ECM molecules were used as described [26]. Briefly, for immunohistochemical investigations, dewaxed and alcohol-hydrated sections underwent blcking of endogenous peroxidase activity by methanol with 0.5% H_2_O_2_. After incubation with 20% goat serum, sections were incubated with specific monoclonal or polyclonal antibodies (Table 2) overnight at 4°C. As negative control, primary antibodies were substituted with either rabbit serum for polyclonal Abs (1:3000; R4505; Sigma Aldrich, Taufkirchen, Germany) or mouse Balb/c serum for monoclonal Abs (1:1000; CBL600; Merck Millipore, Darmstadt, Germany). Biotinylated goat-anti-rabbit IgG (BA-1000), goat-anti-mouse IgG (BA-9200), diluted 1:200 (Vector Laboratories, Burlingame, CA, USA), were used as secondary antibodies. For the antbody decorin, a peroxidase-coupled rabbit anti-goat IgG (DakoCytomation GmbH, Hamburg, Germany, P0449; 1:100) was used as a secondary antibody. Brain tissue of an adult dog as well as the cerebellum of a distemper dog served as positive controls for the various antigens [28]. 3,3’-diaminobenzidine tetrahydrochloride (DAB) with 0.03% H_2_O_2_ served as a chromogen. Finally, sections were slightly counterstained with Mayer`s hemalaun [28].

**Table 2:** Used primary antibodies, supplier, clonality, immunogen, demasking of antigens (pretreatment) and dilution.

Antibodies	Supplier	Catalogue or clone number	Clonality	Immunogen	Demasking	Dilution
**Aggrecan**	Merck Millipore, Darmstadt, Germnay	AB1031	polyclonal, rabbit	GST fusion protein containing AA 1177–1326 of mouse aggrecan	Chondroitinase (2 hrs)	1:100
**Brevican**	BD Bioscience, Heidelberg, Germany	Clone 2, 610894	monoclonal, mouse	AA 232–394 of rat brevican	Microwave treatment/ CB (20 min)	1:200
**Collagen I**	Abcam Ltd., Cambridge, UK	ab21286	polyclonal, rabbit	Collagen I extracted and purified from murine skin	Microwave treatment/ CB (15 min)	1:1200
**Collagen IV**	Acris Antibodies GmbH, Hiddenhausen, Germnay	BP5031	polyclonal, rabbit	Human placental type IV Collagen.	Protease Type XIV (20 min)	1:50
**Decorin**	R&D Systems GmbH, Wiesbaden, Germany	AF1060	polyclonal, goat	NS0-derived recombinant mouse decorin	TRS (20 min)	1:200
**Fibronectin**	Sigma-Aldrich Chemie GmbH, Taufkirchen, Germany	F3648	polyclonal, rabbit	Purified human fibronectin	Microwave treatment/ CB (15 min)	1:1000
**Laminin**	Quartett, Berlin, Germany	QT2120100401	polyclonal, rabbit		Pronase E (20 min)	1:75
**Neurocan**	Merck Millipore, Darmstadt, Germany	Clone 650.24, MAB5212	monoclonal, mouse	Embryonic rat brain proteoglycans	none	1:800
**Factor VIII (Von Willebrand factor)**	Dakocytomation, Hamburg, Germany	A0082	polyclonal, rabbit	Von Willebrand Factor isolated from human plasma	Pronase E (20 min)	1:200
**Phosphacan**	Merck Millipore, Darmstadt, Germnay	Clone 122.2, MAB5210	monoclonal, mouse		Pronase E (20 min)	1:2000
**CDV-NP**	C. Örvell, Sweden	Clone 3991	monoclonal, mouse	nucleoprotein of CDV	none	1:6000

CB = citrate buffer (pH 6.0), TRS = Target Retrieval Solution, CDV = Canine distemper virus; NP = nucleoprotein

The percentage of ECM-positive structures (histochemically and immunohistochemically) and factor VIII expression in white matter areas (lesions and NAWM as well as controls) was evaluated as described [26] Cerebellar sections were photographed with a color video camera (Color View II, 3,3 Megapixel CCD; Soft Imaging System, Münster, Germany) mounted on an Axiophot microscope (Zeiss, Oberkochen, Germany) with a 5x objective. The positive structures were measured interactively after manually outlining the total white matter area using the analySis 3.1 software package (SOFT Imaging System, Münster, Germany; [26]). Data are presented as percentage of the ECM- or factor VIII-positive area in relation to the total lesioned area or investigated white matter area, respectively.

The statistical analysis of the histochemical and immunohistochemical data was carried out by using the statistics program Statistical Analysis System (SAS) for Windows, version 9.1, (SAS Institute Inc., Cary, USA) in the Department of Biometry, Epidemiology and Information Processing of the University of Veterinary Medicine, Hannover, Germany.

Goodness of fit of lognormal data was assessed by an analysis of the model residuals using Q-Q-plots and the Kolmogorov-Smirnov test. Statistical differences among groups were evaluated employing a one-way ANOVA followed by multiple pair-wise comparisons of means with alpha-adjustment by Tukey-Kramer. For all tests, statistical significance was designated as p ≤ 0.05.

Additionally, double labeling of factor VIII immunohistochemistry combined with azan stain was performed on selected slides of chronic distemper lesions. Sections were initially immunostained, followed by subsequent azan stain.

A correlation of positively stained areas in the factor VIII immunohistochemistry and histochemical stains as well as immunohistochemical detetction of ECM molecules, respectively, was performed by calculating the Spearman correlation coefficient using the statistics program SAS.

### Transcriptional analysis of ECM-associated genes

Publically available expression data from a previously published microarray study upon CDV-DL performed on GeneChip canine genome 2.0 arrays (Affymetrix, Santa Clara, USA), accessible via the ArrayExpress database (accession number: E-MEXP-3917; http://www.ebi.ac.uk/arrayexpress) were used to extract the expression values of a manually created gene list [8]. A literature-based list of 410 genes supposed to be related to synthesis and degradation of ECM or fibrosis was manually created [26]. If necessary, previously published murine and human genes, implied in ECM-associated processes were converted into orthologous canine gene symbols using the MADGene web tool [30] (http://cardioserve.nantes.inserm.fr/madtools/madgene/). Furthermore, selected orthologous canine genes were retrieved using Information Hyperlinked over Proteins [31] (http://www.ihop-net.org/UniPub/iHOP/). The original study was performed using RNA isolated from frozen brain sections of twelve control animals (group 1) and 14 CDV-infected dogs suffering from spontaneously occurring and immunohistologically confirmed CDV-DL [8]. All of these animals displayed only one lesion type in the processed brain areas. Based on histopathological findings, the animals were classified into individuals with acute CDV-DL lesions (group 2, n = 5), subacute CDV-DL lesions with demyelination but without inflammation (group 3, n = 6), and chronic CDV DL lesions with demyelination and inflammation (group 4, n = 3; Table 3).

**Table 3:** List of the different groups and numbers of animals per group in microarray and real time quantitative PCR (RT-qPCR) analysis.

Group no.	1	2	3	4
Definition	control	acute CDV leukoencephalitis	subacute CDV leukoencephalitis with demyelination but without inflammation	chronic CDV leukoencephalitis with demyelination and inflammation
No. of animals in microarray analysis	12	5	6	3
No. of animals in RT-qPCR analysis	4	5	5	3

The 410 genes of the literature-based gene list were assigned to a list of 811 Affymetrix probe sets presented on the GeneChip canine genome 2.0 array using NetAffx [32]. Log_2_ transformed, GC-Robust Multiarray Averaging (RMA) preprocessed data sets from the original study were used for further analysis. Statistical testing for differential expression was performed employing the Linear Models for Microarray Data (LIMMA) algorithm with p-value adjustment for multiple testing according to the False Discovery Rate (FDR) algorithm developed by Benjamini and Hochberg employing the Babelomics web-application [33] (Babelomics 5; 2015). The fold change (FC) was calculated as the ratio of the inverse-transformed arithmetic means of the log_2_-transformed expression values. Down-regulations are shown as negative reciprocal values [34]. A FDR ≤ 5% and a FC ≥ 2.0 or ≤ −2.0 was determined to define differentially expressed probe sets (DEPs) [35]. Lists of differentially expressed genes (DEGs) were generated from DEPs by selecting the probe set with the highest significant absolute fold change in one of the comparisons, if the gene was represented by multiple probe sets [36].

### Real time quantitative PCR (RT-qPCR)

Additionally, RT-qPCR was performed to detect mRNA of different MMPs and TIMPs as well as reversion-inducing cysteine-rich protein with Kazal motifs (RECK), respectively. For the RT-qPCR, 8 control dogs without any pathomorphological alterations of the cerebella were used. A total of 26 dogs, spontaneously infected with CDV was investigated. 14 dogs showed acute, 6 subacute non-inflammatory and 6 subacute and chronic inflammatory lesions. All investigated dogs belonged to the population which was also used for microarray analysis (Table 3).

#### RNA isolation and cDNA synthesis

Total RNA was extracted from 200 μm of frozen tissue sections using a silica gel-based membrane (RNeasy Lipid Tissue Mini Kit; Qiagen GmbH, Hilden, Germany), followed by digestion with RNase-Free DNase (Qiagen GmbH, Hilden, Germany) according to the manufacturer’s protocols. The concentration of RNA samples was ascertained by measuring the optical density at 260 nm. Total RNA was reverse transcribed to complementary DNA (cDNA) using the Omniscript kit (Qiagen GmbH, Hilden, Germany) with RNase Out (Invitrogen^™^ GmbH, Hilden, Germany) and Random Primers (Promega GmbH, Mannheim, Germany).

#### Primer design

Primers were designed using the Primer 3 software or the Beacon Designer version 2.1 Software (Premier Biosoft International, Palo Alto, USA). Primers specific for MMP14, TIMP1, TIMP2 and GAPDH were taken from the literature [23]. Primers were purchased from MWG Biotech AG, Ebersberg, Germany.

RT-qPCR and data analysis were performed using the Mx3005P QPCR System (Stratagene Europe, Amsterdam, Netherlands). The reactions were carried out in 8x strip tubes (Stratagene Europe, Amsterdam, Netherlands) covered with Optical Cap, 8x strip (Stratagene Europe, Amsterdam, Netherlands). In addition to the cDNA samples, tenfold serial dilutions of purified, agarose gel extracted (NucleoSpin Extract II kit; Macherey-Nagel GmbH & Co KG, Düren, Germany) RT-PCR products ranging from 10^8^ to 10^2^ copies per sample were used as templates to generate standard curves for estimation of copy numbers in each plate. In addition to the templates, plates contained duplicates of serially diluted samples for the standard curves and a no template control in duplicates. Initial optimization runs were performed to determine the exact composition of the PCR reaction mix (Brilliant SYBR-green QPCR Core Reagent Kit; Stratagene Europe, Amsterdam, Netherlands), reaction time and temperature. The quantitation was carried out in a 25 μl volume using the SYBR-Green I dye.

qPCR with Sybr Green I dye (1:40000) was performed under the following conditions: one initial denaturation step at 95°C for 10 minutes; 95°C for 30 seconds, 57°C (TIMP1, -2) or 60°C (RECK) or 61°C (MMP13) or 63°C (MMP14) or 64°C (MMP2, GAPDH) annealing temperature for 1 minute, and 72°C for 30 seconds; repeated 40 times; and a final extension step at 72°C for 1 minute. The melting curve analysis was performed starting with 95°C for 1 minute followed by 40 cycles starting with 55°C increasing the temperature 1°C per cycle. Amplification was performed using 0,05 U/μl SureStart Taq DNA Polymerase in 1 x Core PCR buffer 10x with 2.5 mM MgCl_2_ (4.0 mM for TIMP1), 8.0% Glycerol, 3% DMSO (4% for TIMP1, -2), 150 nM of each primer (Table 4), 30 nM Rox as reference dye and 200 μM dNTP mix.

**Table 4:** Target gene, sequence, amplicon length and GenBank accession of primers used for RT-qPCR analysis.

Target gene	Sequence of the primer	Amp-licon length [bp]	GenBank accession
MMP2	Forward: 5‘-GGAGATCTTCTTCTTCAAGGACCG-3‘	89	AF177217
MMP2	Reverse: 5‘-AGAATGTGGCTACCAGCAGGG-3	89	AF177217
MMP9	Forward: 5’-CATGACATCTTCCAGTACCAAG-3’	85	AB006421
MMP9	Reverse: 5’-GGTTCACCTCATTCCGAGAA-3’	85	AB006421
MMP9	Probe: 5’-FAM-CTACTTCTGCCAGGACCGCTTCTACT-TAMRA-3’	85	AB006421
MMP12	Forward: 5’-CCCTTTTGATGGCCGAGGTG-3’	117	DQ395095
MMP12	Reverse: 5’-TTTGTGCCTTTGTAGGTTTTAGTCC-3’	117	DQ395095
MMP13	Forward: 5‘-GGCTTAGAGGTCACTGGCAAAC-3‘	118	AF201729
MMP13	Reverse: 5‘-TGGACCACTTGAGAGTTCGGG-3‘	118	AF201729
MMP14	Forward: 5‘-GATTCCTTCCCAGACCTTGATGTTT-3‘	116	AY534615
MMP14	Reverse: 5‘-GGATGCCCAATGGAAAGACCTAC-3‘	116	AY534615
TIMP1	Forward: 5‘-ACGGACACTTGCAGATCAAC-3‘	94	AF077817
TIMP1	Reverse: 5‘-GCAGCATAGGTCTTGGTGAA-3‘	94	AF077817
TIMP2	Forward: 5‘-CCATCAAGCGGATTCAGT-3‘	89	AF095638
TIMP2	Reverse: 5‘-GGAAGGAGCCGTGTAGATAA-3‘	89	AF095638
RECK	Forward: 5‘-CATCTGTGGGCACAATGGGG-3‘	81	AB110699
RECK	Reverse: 5‘-GGCCCGTAGTAATCGACTGC-3‘	81	AB110699
GAPDH	Forward: 5‘-GTCATCAACGGGAAGTCCATCTC-3‘	84	AB038240
GAPDH	Reverse: 5‘-AACATACTCAGCACCAGCATCAC-3‘	84	AB038240

bp = base pairs; MMP = matrix metalloproteinase; TIMP = tissue inhibitor of matrix-metalloproteinases; RECK = reversion-inducing-cysteine-rich protein with Kazal motifs; GAPDH = glyceraldehyd-3-phosphat-dehydrogenase

qPCR for MMP-9 was performed using 0.025 U/μl SureStart Taq DNA Polymerase in 1 x Core PCR buffer 10x with 5.0 mM MgCl_2_, 300 nM of each primer, 200 nM TaqMan probe, 76 nM Rox as reference dye and 200 μM dNTP mix. Amplification was performed under the following temperature conditions: one initial denaturation step at 95°C for 10 minutes; 95°C for 15 seconds and 60°C for 1 minute; repeated 40 times.

For comparison, gene expressions were normalized against the housekeeping gene GAPDH (relative expression), after a separate calculation for GAPDH. The relative percentage of target specific gene expression was calculated as follows: X / Y x 100 = normalized target specific gene expression (X = target specific gene expression level; Y = housekeeping gene (GAPDH) expression level).

#### Correlation of expression values obtained by RT-qPCR and microarray analysis

Expression values obtained by RT-qPCR and microarray analysis were compared on a gene-by-gene level by Spearman correlation using SPSS (IBM SPSS Statistics, Version 21, IBM, Chicago, IL, USA) for *MMP2*, *MMP9*, *MMP13*, *MMP14*, *TIMP1*, *TIMP2* and *RECK*. For *MMP12* no probeset was present on Affymetrix GeneChip canine genome 2.0 array. The input data into the correlation analysis were GC-RMA normalized log_2_-transformed expression values for the microarray analysis and GAPDH-normalized expression ratios for RT-qPCR, respectively.

Direct comparision of RNA-expression values obtained from the same specimen detected either by microarray analysis or RT-qPCR was possible only in a subset of animals (control: n = 4; acute: n = 5; subacute non-inflammatory: n = 5; subacute and chronic inflammatory: n = 3).

## Results

### Progressive deposition of ECM in CDV lesions

**Fig 1:**
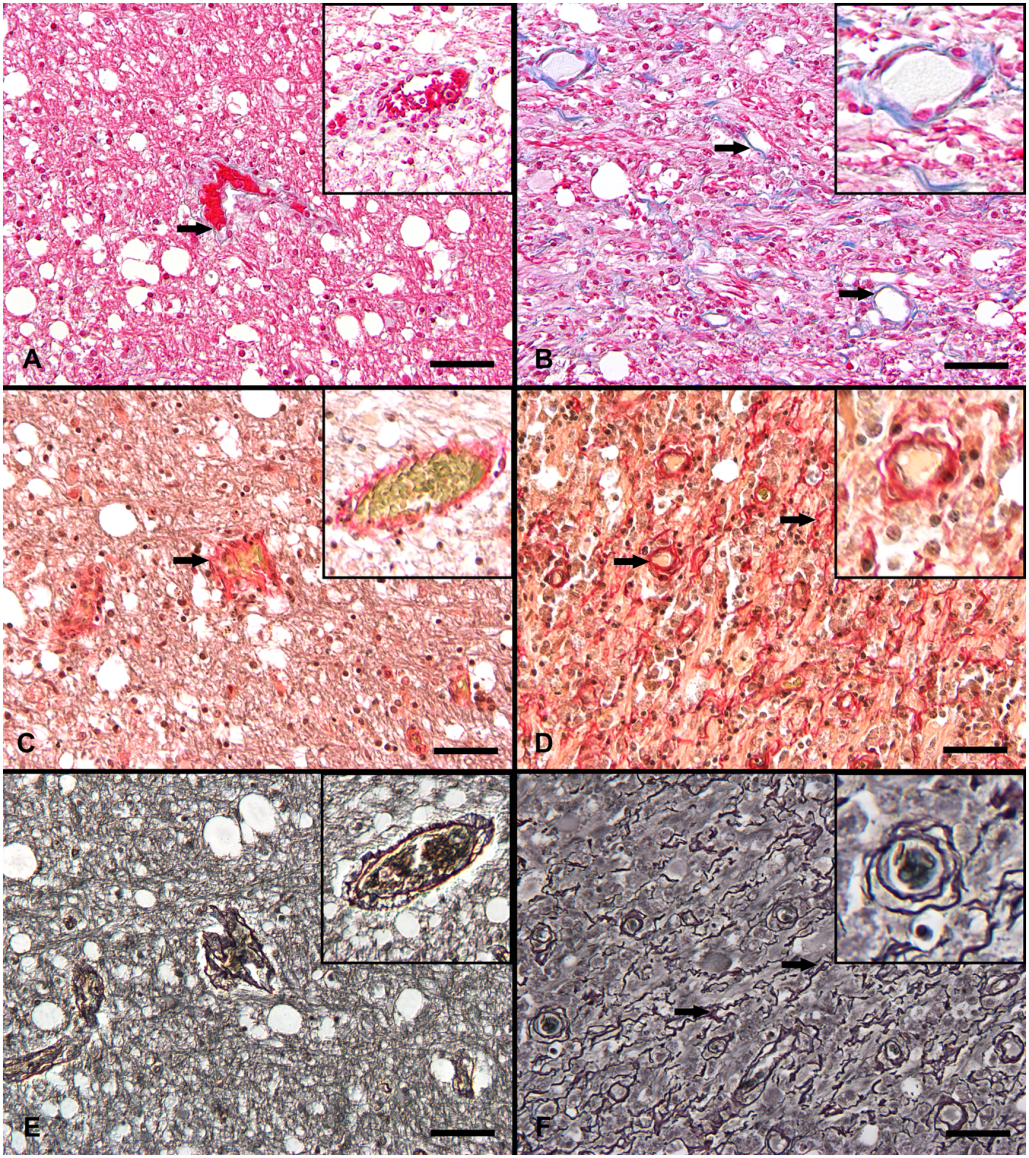
Histochemical staining of CDV-infected canine brains. **A**: Cerebellum, white matter, acute lesion with azan-positive vascular walls (arrow). **B**: Cerebellum, white matter, chronic lesion, extensive reticular, extracellular, intralesional deposition of azan-positive blue material (arrows). Azan stain. **C**: Cerebellum, white matter, acute lesion, dark red reaction of vascular wall (arrow). Modified picrosirius red stain. **D:** Cerebellum, white matter, chronic lesion, intralesional grid-like picrosirius red reaction (arrows). Modified picrosirius red stain. **E**: Cerebellum, white matter, acute lesion, black signal around blood vessels. Gomori`s silver stain. **F**: Cerebellum, white matter, chronic lesion, branched filamentous reaction in the center of the lesion and around blood vessels (arrows). Gomori`s silver stain. **A-F:** Insets show vascular structures in a higher magnification. All scale bars = 50 μm.

In the azan stain, the canine cerebella of all groups showed a blue staining of meningeal extracellular substance as well as meningeal and parenchymal blood vessels (S1 Fig and Fig 1). Additionally, in group 8, filamentous extracellular structures associated with the vessels and astrocytes of predominantly low to moderate intensity, centrally located in the demyelinating lesion were seen (Fig 1). The quantitative evaluation of group 1 to 7 showed a geometric mean of 0.019% to 0.024% of azan-positive area compared to the investigated white matter area. Furthermore in group 8, there was an increase of the azan-positive reaction with a geometric mean of 0.18% (Table 5). Group 1 to 7 showed a statistically significant difference of azan-positive reaction compared to group 8 (Table 6).

**Table 5:** Morphometrically determined areas of extracellular matrix molecule deposition in the cerebella of CDV-infected dogs and controls (positive area per total white matter area [%]).

Parameter	Group 1	Group 2	Group 3	Group 4	Group 5	Group 6	Group 7	Group 8
Azan	0.019 (0.015;0.024)	0.019 (0.009;0.040	0.027 (0.015;0.053)	0.030 (0.017;0.053)	0.040 (0.018;0.092)	0.035 (0.017;0.073)	0.024 (0.009;0.063)	0.180 (0.157;0.206)
PSR, red color	0.059 (0.041;0.084)	0.042 (0.018;0.098)	0.067 (0.024;0,191)	0.047 (0.020;0.114)	0.047 (0.021;0.103)	0.099 (0.028;0.351)	0.254 (0.084;0.767)	1.100 (1.032;1.173)
PSR, blue color	0.028 (0.028;0.029)	0.027 (0.013;0.060)	0.059 (0.017;0.206)	0.095 (0.012;0.732)	0.108 (0.026;0.442)	0.095 (0.036;0.254)	0.050 (0.010;0.250)	0.614 (0.279;1.351)
Gomori	0.002 (0.001;0.004)	0.003 (0.002;0.004)	0.005 (0.001;0.016)	0.002 (0.001;0.005)	0.005 (0.002;0.013)	0.006 (0.001;0.026)	0.003 (0.001;0.009)	3.555 (0.164;7.295)
Aggrecan	0.012 (0.006;0.024)	0.031 (0.018;0.055)	0.072 (0.037;0.142)	0.148 (0.059;0.369)	0.098 (0.062;0.157)	0.131 (0.075;0.229)	0.133 (0.075;0.236)	0.185 (0.108;0.319)
Type I collagen	0.277 (0.174;0.440)	0.375 (0.244;0.577)	0.489 (0.301;0.795)	0.464 (0.355;0.607)	0.720 (0.430;1.204)	1.069 (0.398;2.870)	1.487 (0.788;2.808)	5.515 (4.952;6.141)
Type IV collagen	0.348 (0.177;0.685)	0.200 (0.086;0.466)	0.552 (0.244;1.248)	0.724 (0.344;1.525)	0.727 (0.391;1.350)	0.971 (0.485;1.945)	1.034 (0.748;1.432)	1.725 (1.248;2.385)
Fibronectin	1.012 (0.795;1.288)	0.764 (0.454;1.286)	0.692 (0.378;1.266)	1.192 (0.438;3.242)	1.674 (1.208;2.320)	1.873 (1.199;2.925)	1.582 (0.936;2.674)	3.942 (3.493;4.449)
Laminin	0.378 (0.235;0.608)	0.344 (0.210;0.566)	0.478 (0.198;1.156)	0.520 (0.224;1.209)	0.620 (0.351;1.096)	1.417 (0.643;3125)	1.217 (0.805;1.839)	n. d.
Phosphacan	6.273 (5.782;6.806)	4.734 (3.438;6.519)	4.071 (2.885;5.743)	4.757 (2.854;7.930)	3.300 (2.765;3.936)	2.429 (1.540;3.830)	3.663 (2.153;6.233)	2.261 (2.102;2.431)
Factor VIII	1.310 (1.025;1.674)	0.717 (0.264;1.945)	0.809 (0.308;2.124)	0.841 (0.323;2.188)	1.127 (0.519;2.449)	1.913 (1.225;2.989)	1.579 (1.373;1.815)	2.281 (1.560;3.335)

Highlighted areas showed a statistically significant (p ≤ 0.05) up- (red) or down-regulation (green) compared to the controls (group 1). 1 = control group; 2 = normal appearing white matter (NAWM); 3 = antigen without lesion; 4 = vacuolation; 5 = acute; 6 = subacute without inflammation; 7 = subacute with inflammation; 8 = chronic. PSR = picrosirius red stain; n. d. = not detectable. Data are given as percentage of positively labeled area per total white matter area (geometric mean [slg = geometric mean—lower geometric standard deviation; sug = geometric mean + upper geometric standard deviation]).

**Table 6:** p-values of the multiple pair-wise comparisons of geometric means (Tukey-Kramer test) of histochemical stains (logarithmic data).

Group	L Azan	L PSR-red	L PSR-blue	L Gomori
**1 and 2**	0.9806	0.5102	0.8815	0.6679
**1 and 3**	0.3499	0.7410	0.2962	0.2096
**1 and 4**	0.4015	0.7311	0.3699	0.9622
**1 and 5**	0.0843	0.7129	0.1128	0.1728
**1 and 6**	0.1545	0.4421	0.1105	0.1417
**1 and 7**	0.5767	0.0157	0.4825	0.7420
**1 and 8**	0.0004	0.0028	0.0016	<.0001
**2 and 3**	0.1855	0.1078	0.0566	0.3235
**2 and 4**	0.3010	0.7804	0.1744	0.7348
**2 and 5**	0.0224	0.6803	0.0076	0.2626
**2 and 6**	0.0604	0.0412	0.0097	0.2072
**2 and 7**	0.4617	<.0001	0.2115	0.9173
**2 and 8**	<.0001	<.0001	0.0001	<.0001
**3 and 4**	0.9190	0.4046	0.9865	0.2754
**3 and 5**	0.2212	0.2846	0.3494	0.8571
**3 and 6**	0.4298	0.4802	0.3486	0.6976
**3 and 7**	0.7137	0.0026	0.6543	0.2916
**3 and 8**	0.0006	0.0011	0.0022	<.0001
**4 and 5**	0.4315	0.9749	0.5220	0.2300
**4 and 6**	0.6313	0.2068	0.5095	0.1910
**4 and 7**	0.7123	0.0030	0.7230	0.8002
**4 and 8**	0.0025	0.0009	0.0079	<.0001
**5 and 6**	0.7117	0.1207	0.9588	0.8290
**5 and 7**	0.1844	0.0006	0.2206	0.2392
**5 and 8**	0.0048	0.0003	0.0093	<.0001
**6 and 7**	0.3233	0.0205	0.1912	0.1874
**6 and 8**	0.0027	0.0037	0.0082	<.0001
**7 and 8**	0.0006	0.1512	0.0011	<.0001

White feld = significant (p ≤ 0.05); gray field = not significant; 1 = control group; 2 = normal appearing white matter (NAWM); 3 = antigen without lesion; 4 = vacuolation; 5 = acute; 6 = subacute without inflammation; 7 = subacute with inflammation; 8 = chronic; PSR-red = picrosirius red stain, red signal; PSR-blue = picrosirius red stain, blue signal; Gomori = Gomori silver stain, L = logarithmic data

In the modified picrosirius red stain, the meninges and blood vessels in the cerebellum of the control dogs (group 1) showed a dark red signal, whereas the non-vessel associated part of the white matter was pale greenish (S1 Fig). A similar reaction pattern was found in the cerebellum of CDV-infected dogs in group 2 (NAWM). Within distemper lesions, this staining produced two different patterns in some sections. Therefore, the different positive signals—classified as red color indicating collagens and blue color indicating carboxylated mucosubstances like hyaluronan—were evaluated separately. In group 3 to 7, the extracellular blue signal was widely distributed within the lesions in a reticular pattern, whereas the extracellular red signal was similar to control brain tissue (Fig 1). In contrast, in group 8, the expression of the blue signal was mainly restricted to the edge of lesions. Additionally, a dark red, filamentous, extracellular net-like reaction pattern was detectable, which was localized in the center of the lesions (Fig 1). This signal showed a yellow-orange to purple birefringence in polarized light.

In group 8, the geometric mean of the blue signal related to the investigated white matter area was 0.614%, whereas in the other groups it ranges from 0.027% to 0.108% (Table 5). Group 1 to 7 showed a statistically significant difference of the blue signal produced by the picrosirius red stain compared to group 8 (Table 6).

An increase of the geometric mean of the morphometrically identified positive red signal of 1.1% was found in group 8 compared to a range of geometric means of 0.042% to 0.254% of group 1 to 7 (Table 5). Groups 1, 3, 4, 5, 6 showed a statistically significant difference compared to group 7 and 8 (Table 6).

In the Gomori`s silver stain, the cerebella of control and distemper dogs revealed positive extracellular structures in the gray and white matter consisting of a fine granular gray color of varying staining intensity (S1 Fig and Fig 1). Additionally, lesions of group 8 showed an extracellular, reticular to branched, black staining, which was centrally located within demyelinated areas (Fig 1).

An increase in the geometric mean value up to 3.555% was detected in group 8, whereas the other groups showed means of up to 0.006% (Table 5). Group 1 to 7 showed statistically significant differences compared to group 8 (Table 6).

**Fig 2:**
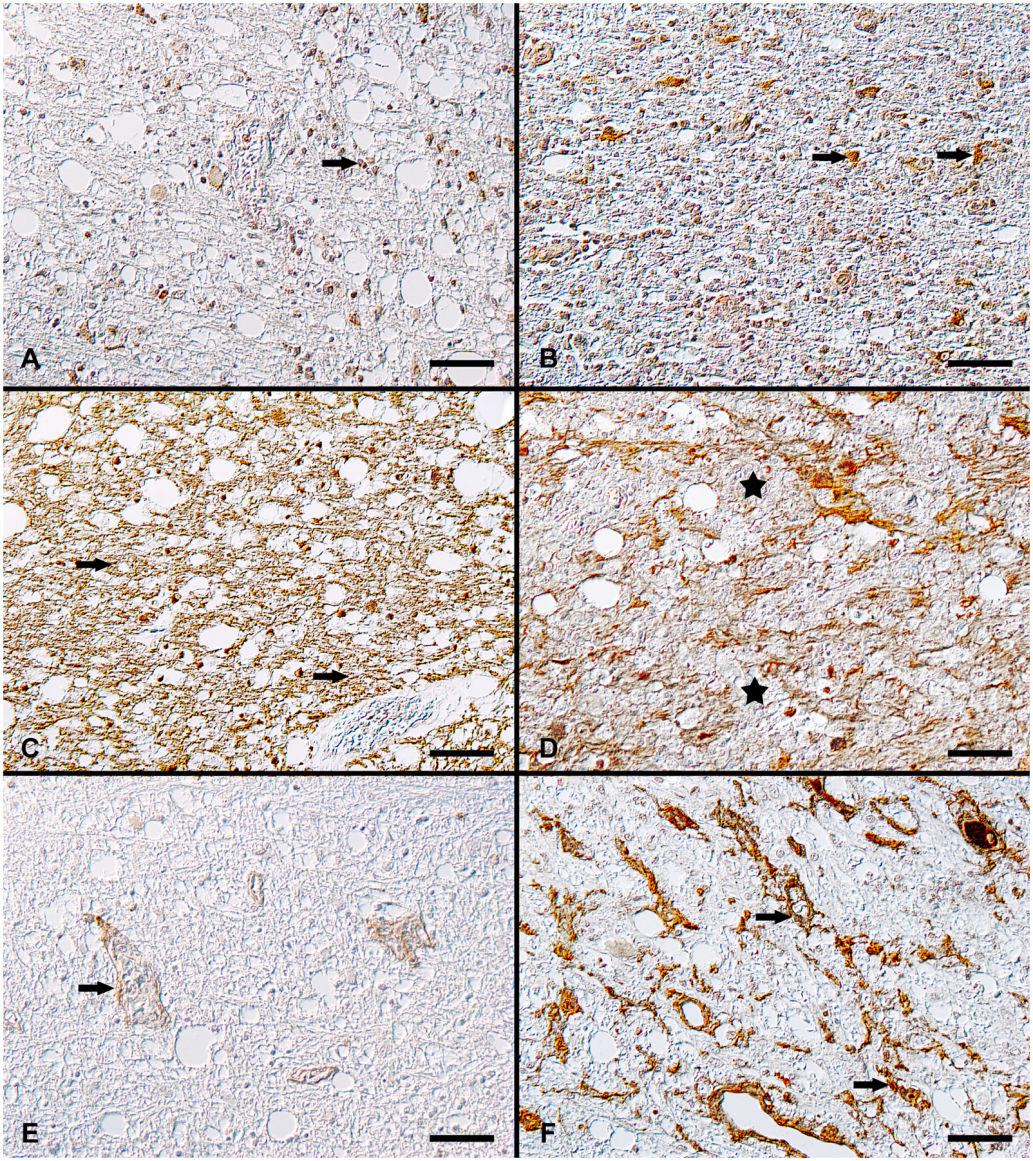
Immunohistochemical detection of aggrecan, phosphacan and fibronectin in CDV-infected canine brains. **A**: Cerebellum, white matter, acute lesion, minimal to mild extracellular expression of aggrecan as well as in the cytoplasm of glial cells (arrow). **B**: Cerebellum, white matter, chronic lesion, prominent intralesional aggrecan deposition mainly in macrophages (arrows). Aggrecan immunohistochemistry (DAB). **C**: Cerebellum, white matter, acute lesion, prominent phosphacan immunoreactivity (arrows). **D**: Cerebellum, white matter, chronic lesion, with moderate to severe reduction of phosphacan immunoreactivity within the lesion (asterisks) corresponding to perivascular inflammatory cell infiltration. Phosphacan immunohistochemistry (DAB). **E**: Cerebellum, white matter, acute lesion, mild expression of fibronectin in vascular walls (arrow). **F**: Cerebellum, white matter, chronic lesion, extensive intralesional reticular extracellular deposition of fibronectin throughout the lesion (arrows). Fibronectin immunohistochemistry (DAB). All scale bars = 50 μm.

In the cerebellum of CDV areas from group 3 to 7 an intralesional extracellular aggregation of small brown aggrecan-positive granules was detected (Fig 2). In the center of chronic plaques (group 8), there was an overall decrease in aggrecan expression, however an accumulation of aggrecan-positive material in foamy macrophages at the edge of the lesions was also detected (Fig 2).

Quantitatively, the geometric means of the aggrecan-positive area related to the total investigated white matter area showed a range of 0.012% in controls (group 1) up to 0.185% in chronic lesions (group 8; Table 5). Group 1 showed a statistically significant lower aggrecan expression compared to group 2 to 8. (Table 7).

**Table 7:** p-values of the multiple pair-wise comparisons of geometric means (Tukey- Kramer test) immunohistochemistry (logarithmic data).

Group	L Agg	L Col-I	L Col-IV	L FN	L LN	L Ph	L FVIII
**1 and 2**	0.0076	0.3255	0.1638	0.3701	0.8164	0.1610	0.1836
**1 and 3**	<.0001	0.0650	0.2356	0.2205	0.5621	0.0281	0.2465
**1 and 4**	<.0001	0.1265	0.1276	0.4985	0.4485	0.3106	0.2878
**1 and 5**	<.0001	0.0056	0.0727	0.1234	0.2199	0.0033	0.9604
**1 and 6**	<.0001	0.0003	0.0172	0.0751	0.0040	<.0001	0.4636
**1 and 7**	<.0001	<.0001	0.0193	0.1486	0.0134	0.0124	0.9550
**1 and 8**	<.0001	<.0001	0.0091	0.0028	-	0.0010	0.2086
**2 and 3**	0.0004	0.1720	0.0005	0.5968	0.2431	0.2233	0.6717
**2 and 4**	<.0001	0.3469	0.0019	0.0699	0.2397	0.8376	0.8291
**2 and 5**	<.0001	0.0063	0.0001	0.0009	0.0497	0.0172	0.0162
**2 and 6**	<.0001	<.0001	<.0001	0.0004	0.0002	<.0001	0.0003
**2 and 7**	<.0001	<.0001	<.0001	0.0030	0.0015	0.0802	0.0456
**2 and 8**	0.0004	<.0001	0.0002	<.0001	-	0.0045	0.0025
**3 and 4**	0.0194	0.9893	0.4571	0.0306	0.7175	0.2879	0.9516
**3 and 5**	0.1651	0.1051	0.3089	0.0002	0.3246	0.1454	0.0300
**3 and 6**	0.0276	0.0025	0.0563	0.0001	0.0014	0.0008	0.0006
**3 and 7**	0.0495	0.0001	0.0665	0.0011	0.0101	0.3936	0.0774
**3 and 8**	0.0567	<.0001	0.0285	<.0001	-	0.0196	0.0041
**4 and 5**	0.1964	0.2335	0.9845	0.3997	0.7110	0.0470	0.1133
**4 and 6**	0.5371	0.0210	0.4760	0.2644	0.0232	0.0009	0.0111
**4 and 7**	0.5362	0.0021	0.4487	0.4360	0.0602	0.1211	0.1568
**4 and 8**	0.9001	<.0001	0.1431	0.0098	-	0.0083	0.0118
**5 and 6**	0.4069	0.1242	0.3811	0.7011	0.0172	0.0403	0.1679
**5 and 7**	0.4714	0.0112	0.3682	0.9984	0.0665	0.6942	0.9876
**5 and 8**	0.2647	<.0001	0.1093	0.0223	-	0.1287	0.0905
**6 and 7**	0.9695	0.2146	0.9223	0.7224	0.7863	0.0250	0.2182
**6 and 8**	0.5351	0.0006	0.2778	0.0343	-	0.8338	0.3541
**7 and 8**	0.5298	0.0073	0.3211	0.0228	-	0.0824	0.0976

White field = significant (p≤0.05); gray feld = not significant; 1 = control group; 2 = normal appearing white matter; 3 = antigen without lesion; 4 = vacuolation; 5 = acute; 6 = subacute without inflammation; 7 = subacute with inflammation; 8 = chronic group; IHC = immunohistochemistry; Agg = Aggrecan; Col-I = Collagen I; Col-IV = Collagen IV; F VIII = Factor VIII; FN = Fibronectin; LM = Laminin; Ph = Phosphacan; L = logarithmic data

In group 6 and 7 (subacute lesions without and with inflammation), there was a mild reduction of the phosphacan-positive area compared to the controls and early lesions. In group 8 (chronic lesions), a moderate to severe reduction of the phosphacan immunoreactivity was detected. Moreover, an accumulation of phosphacan-positive deposits in foamy macrophages was seen (Fig 2).

Quantitatively, a geometric mean of the phosphacan-positive area related to the investigated white matter area in group 1 (controls) of 6.273% was detected. The geometric mean decreased down to 2.261% in group 8 (chronic lesions), respectively (Table 5).

In the statistical analysis, group 3, 5, 6, 7 and 8 showed a statistically significant lower phosphacan expression compared to group 1. Early lesions (group 2 and 3) differed also significantly from advanced lesions (group 6 and 8; Table 7).

The fibronectin expression in control dogs (group 1) and CDV-infected animals appeared as a diffuse cytoplasmic signal of neurons and glial cells (S2 Fig). In addition, a fibronectin labeling was detected in the meningeal and parenchymal blood vessels (Fig 2). Additionally, in chronic lesions of group 8, there was a finely granular to densely branched, extracellular distribution of fibronectin immunoreactivity in the demyelinating areas. In addition, a intracytoplasmic signal was found in macrophages (Fig 2).

Quantitatively, there were geometric means of 0.692% to 1.873% in group 1 to 7. There was an increase of the positive area related to the total lesioned area up to a geometric mean of 3.942% in the chronic lesions of group 8 (Table 5).

In the statistical analysis, controls and NAWM (group 1 and 2) showed a statistically significant lower type I collagen expression compared to group 5, 6, 7 and 8 (Table 7).

**Fig 3:**
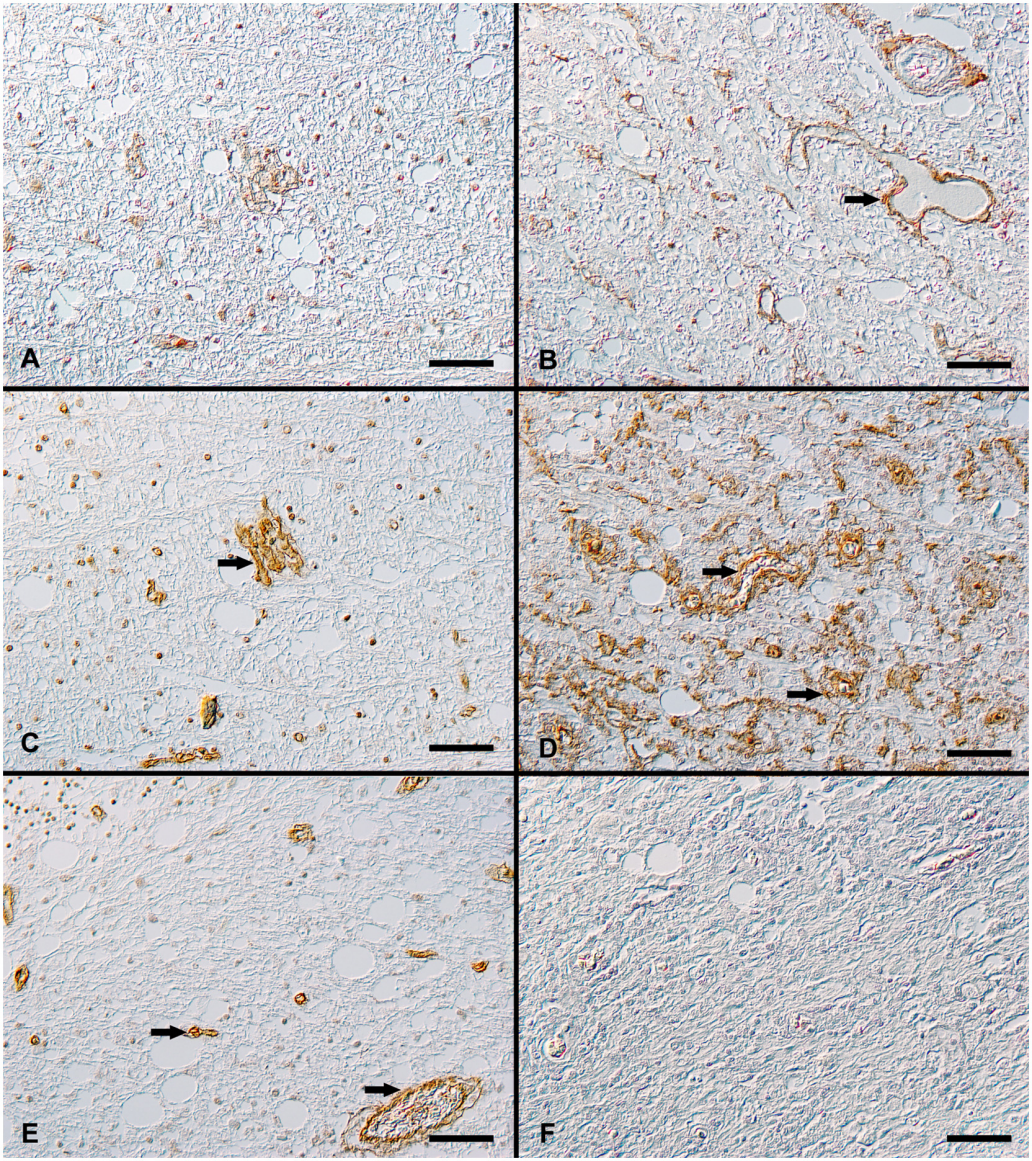
Immunohistochemical detection of collagen type I and IV as well as laminin in CDV-infected canine brains. **A**: Cerebellum, white matter, acute lesion, mild to moderate type I collagen expression associated with basement membranes. **B**: Cerebellum, white matter, chronic lesion, extracellular deposition of type I collagen, associated with vascular walls (arrow). Type I collagen immunohistochemistry (DAB). **C**: Cerebellum, white matter, acute lesion, mild to moderate type IV collagen expression around basement membranes of vascular walls (arrow). **D**: Cerebellum, white matter, chronic lesion, extensive extracellular deposition of type IV collagen (arrows) and intracellular type IV collagen detection in macrophages. Type IV collagen immunohistochemistry (DAB). **E**: Cerebellum, white matter, acute lesion, laminin expression is associated with basement membranes of blood vessels (arrow). **F**: Cerebellum, white matter, chronic lesion, with nearly complete absence of laminin immunoreactivity in the demyelinating lesion. Laminin immunohistochemistry (DAB). All scale bars = 50 μm.

Type I collagen expression in acute and subacute CDV lesions (group 5 to 7), consisted of an intralesional, extracellular signal associated with vascular walls (Fig 3). In the chronic lesions (group 8), an extension of this reaction with a reticular distribution pattern was observed in the center of demyelinating lesions. The subendothelial space and the basement membrane of medium-sized vessels also showed a strong signal (Fig 3).

Quantitatively, there were geometric means of 0.277% to 1.487% in group 1 to 7. An increase in the geometric mean of the positive area within the white matter of 5.515% was identified in the chronic lesions (group 8; Table 5).

In the statistical analysis, group 8 showed a significantly increase in Type I collagen expression compared to all other groups (Table 7).

Immunohistochemical expression of type IV collagen antigen was found in the control group and the CDV-infected brains as a medium to dark brown signal in vascular basement membranes and meninges (S2 Fig and Fig 3). Furthermore, in group 8 (chronic lesions), a light brown, extracellularly located signal within demyelinating lesions and also positive randomly distributed cells with the morphology of macrophages were recognized (Fig 3).

Quantitatively, the geometric mean of the type IV collagen-positive area related to the total lesion area in group 1 to 5 ranged from 0.2% to 0.727%. There was a slight increase to a geometric mean of 0.971% to 1.725% in group 6 to 8 (Table 5).

In the statistical analysis, subacute and chronic lesions (group 6 to 8) showed an increase in type IV collagen expression compared to group 1 and 2 (Table 7).

Laminin expression was detected in the leptomeninges and in the vascular basement membrane within the meninges and the parenchyma in controls (group 1) and in CDV-infected dogs of group 2 to 7 (S2 Fig and Fig 3). In contrast, the expression of laminin in chronic demyelinating lesions (group 8) was nearly completely absent (Fig 3).

Quantitatively, the geometric means of the positive area in group 1 to 5 ranged from 0.344% to 0.62%. The geometric mean of the laminin-positive area related to total lesioned area of the white matter increased in group 6 (geometric mean of 1.417%). In group 7, a decline with a geometric mean of 1.217% was noticed (Table 5)

In the statistical analysis, subacute lesions (group 6 and 7) showed a statistically significant increase in laminin expression compared to group 1 to 3 (Table 7).

In control dogs, factor VIII expression was detected in endothelial cells and to a lesser extent in the subendothelial matrix of the vessel wall. In the cerebellum of CDV-infected dogs, the same reaction pattern was observed (S3 Fig). Additionally, in subacute and chronic lesions, a mild overall increase in factor VIII expression was noted. Conversely, some chronic lesions showed a decrease of the signal in the center of the lesion.

Quantitatively, the geometric mean of the factor VIII-positive area related to the total lesioned area in group 1 to 5 ranged from 0.717% to 1.310%. There was a slight increase to a geometric mean of 1.579%, 1.913% and 2.281% in group 7, 6 and 8, respectively (Table 5).

In the statistical analysis, group 6 and 8 showed a statistically significant difference in factor VIII expression compared to group 2, 3 and 4. Group 7 revealed significant differences compared to group 2. In addition, group 5 differed significantly from group 2 and 3 (Table 7).

By using the factor VIII/azan stain double labeling, factor VIII expression was found in endothelial cells of capillaries or in the subendothelial matrix of large blood vessels. Azan-positive signals were located in close proximity to blood vessels but also throughout the whole chronic lesion (S3 Fig).

With the Pearson’s correlation coefficient, factor VIII expression showed a weakly positive correlation with the red signal of the picrosirius red stain (*r = 0*.*299*, *p* = 0.024) and with aggrecan (*r* = 0.308, p = 0.019), type I collagen (*r* = 0.315, p = 0.018), fibronectin (*r* = 0.288, p = 0.031), laminin (*r* = 0.387, p = 0.006) as well as type IV collagen (*r* = 0.517, p = <0.0001).

Neither in the cerebella of control dogs nor in any lesion type of CDV-infected dogs, brevican, decorin and neurocan expression was detected. In case of neurocan, there was a mild reaction in the spinal nerve roots and perineuronal structures (endo- and perineurium) in three animals.

To further investigate the mRNA expression of ECM molecules and their related proteins for degradation and synthesis, an analysis of a list of genes extracted from a previously published microarray study [8] was performed.

### Transcriptional changes of ECM-related genes in CDV-infected dogs

**Fig 4:**
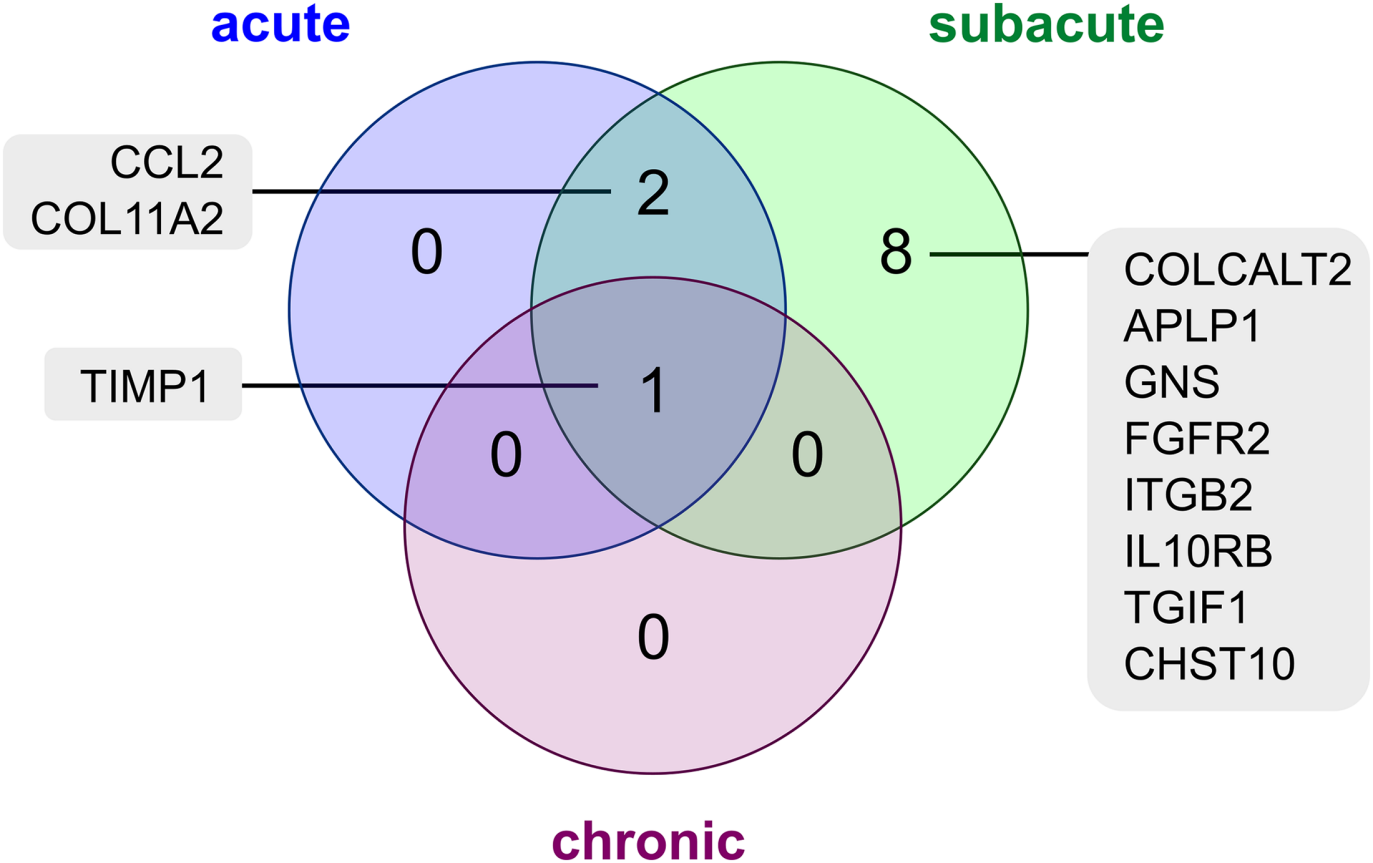
Venn diagram of differentially expressed ECM-associated genes in canine distemper. The Venn diagram displays the intersection of differentially expressed genes associated with the synthesis and degradation of extracellular matrix in acute, subacute, and chronic canine distemper lesions as obtained by re-analysis of a previously published microarray study (ArrayExpress E-MEXP-3917)[8].

A total of 11 genes (2.7%), namely *collagen beta(1-O)galactosyltransferase 2 (COLALT2)*, *amyloid beta (A4) precursor-like protein 1 (APLP1)*, *glucosamine (N-acetyl)-6-sulfatase (GNS)*, *fibroblast growth factor receptor 2 (FGFR2)*, *integrin*, *beta 2 (complement component 3 receptor 3 and 4 subunit) (ITGB2)*, *TIMP metallopeptidase inhibitor 1 (TIMP1)*, *chemokine (C-C motif) ligand 2 (CCL2)*, *interleukin 10 receptor*, *beta (IL10RB)*, *TGFB-induced factor homeobox 1 (TGIF1)*, *collagen*, *type XI*, *alpha 2 (COL11A2)*, and *carbohydrate sulfotransferase 10 (CHST10)* were found to be differentially expressed in at least one comparison in the literature-based gene list comprised of 410 genes (S1 Table) associated with synthesis and degradation of ECM or fibrosis present on Affymetrix GeneChip canine genome 2.0 array (Table 8). Compared to control dogs, 6 genes (*TIMP1*, *CCL2*, *GNS*, *ITGB2*, *IL10RB*, *TGIF1*) were upregulated, 5 genes (*COL11A2*, *COLGALT2*, *APLP1*, *FGFR2*, *CHST10)* were downregulated (Table 8). 3, 11, and 1 genes were detected to be differentially expressed in acute, subacute and chronic CDV-infection, respectively (Fig 4). *TIMP1* showed a differential expression in all stages of the disease with increasing expression values from acute to chronic lesions (Fig 4; Table 8).

**Table 8:** List of differentially expressed genes (DEGs) by microarray analysis.

Gene Symbol	Gene Title	Adj. p-value			Fold change		
		acute vs. control	subacute vs. control	chronic vs. control	acute vs. control	subacute vs. control	chronic vs. control
TIMP1	TIMP metallopeptidase inhibitor 1	0.03	0.00	0.00	5.27	15.98	19.45
CCL2	chemokine (C-C motif) ligand 2	0.01	0.00	0.11	11.98	14.61	8.28
COL11A2	collagen, type XI, alpha 2	0.03	0.00	0.17	-2.56	-3.49	-2.35
COLGALT2	collagen beta(1-O)galactosyltransferase 2	0.11	0.01	0.22	-2.09	-2.39	-2.13
APLP1	amyloid beta (A4) precursor-like protein 1	0.98	0.01	0.77	-1.18	-2.18	-1.38
GNS	glucosamine (N-acetyl)-6-sulfatase	0.07	0.02	0.47	2.53	2.35	1.66
FGFR2	fibroblast growth factor receptor 2	0.68	0.03	1.00	-2.16	-4.92	1.07
ITGB2	integrin, beta 2 (complement component 3 receptor 3 and 4 subunit)	0.31	0.04	0.22	2.91	3.36	3.83
IL10RB	interleukin 10 receptor, beta	0.15	0.01	0.32	2.18	2.91	1.85
TGIF1	TGFB-induced factor homeobox 1	0.70	0.02	0.18	1.54	2.67	2.90
CHST10	carbohydrate sulfotransferase 10	0.70	0.01	1.00	-1.34	-2.18	-1.02

Highlighted areas showed a statistically significant up- (red) or down-regulation (green).

With *TIMP1* being the gene with the highest fold change in nearly all CDV groups, a RT-qPCR was performed in order to confirm the results of the changes in TIMP mRNA expression in the microarray analysis. Furthermore, TIMP2 and several MMPs were investigated.

### Transcriptional changes of MMPs and TIMPs in RT-qPCR

The number of *TIMP1* mRNA transcripts varied markedly between the different groups. The highest number was demonstrated in the subacute group. There was a significant up-regulation in all infected groups compared to controls (p ≤ 0.05). There was also a significant up-regulation from acute to subacute lesions (p ≤ 0.05). *TIMP2* mRNA transcripts were similar between the different groups (p > 0.05). The number of *RECK* mRNA transcripts showed a significant up-regulation between controls and subacute lesions (p ≤ 0.05), without significant changes between the other lesion types. *MMP2* mRNA transcripts were down-regulated in acute lesions compared to controls (p ≤ 0.05). *MMP9* mRNA was not detectable in some animals of each group (control group: 1/8; acute group: 3/14; subacute group: 1/6; chronic group: 1/6). The highest number of *MMP9* transcripts was observed in the subacute group, and all infected animals showed higher numbers than control animals (p ≤ 0.05). The number of *MMP12* mRNA transcripts was significantly higher in infected animals compared to controls (p ≤ 0.05). The highest number was present in brains with subacute, non inflammatory lesions followed by a down-regulation in inflammatory, chronic plaques. *MMP13* and *MMP14* mRNA transcripts were neither significantly changed in infected and non-infected animals nor in the different lesion types. There were no significant changes in the *GAPDH* mRNA expression between the four different groups (p > 0.05).

#### High correlation of expression values obtained by RT-PCR and microarray analysis

A very high and high correlation existed between the transcript expression obtained by microarray analysis and the results retrieved by RT-qPCR for *TIMP1* (*r* = 0.98) and *MMP2* (*r* = 0.82). Moderate correlation was found for *MMP9* (*r* = 0.68). For *MMP13* (*r* = 0.26), *MMP14* (*r* = 0.08), *TIMP2* (*r* = 0.45) and *RECK* (*r* = 0.32) low to no correlation was detectable.

## Discussion

The aim of the present investigation was to describe the quantity and distribution of ECM and its spatio-temporal developmental patterns in controls and CDV-infected canine brains. Therefore, a multidirectional approach on the mRNA and protein level using histochemistry, immunohistochemistry, RT-qPCR and microarray analysis was chosen. It should be emphasized that the histochemical/immunohistochemical and the microarray analysis and RT-qPCR data, respectively, are based on differing group assignments and were performed on different populations of study subjects, thus preventing direct comparisons and correlation analyses of genes and proteins.

In summary, there was an accumulation of ECM molecules as demonstrated by different histochemical stains, especially in advanced CDV lesions. These intralesional deposits were further characterized by immunohistochemistry and revealed statistically significant higher accumulation of several ECM molecules namely aggrecan, fibronectin, type I and IV collagen and laminin in CDV lesions compared to controls. In contrast, phosphacan expression was significantly lower in diseased brains compared to control tissue.

The vast majority of accumulated ECM substances in demyelinating CDV-infected lesions consisted of collagen fibers, proteoglycans and glycoproteins. The reactive production and the accumulation of those ECM molecules, mainly of the chondroitin sulfate type, have been described in various experimental nervous system lesions and demyelinating diseases. Their inhibitory role upon axonal regeneration and remyelination as well as their hindrances of CNS regeneration are discussed [37,38]. The reduction of axonal density and the accumulation of damaged axons especially in chronic distemper lesions as well as astrocytic gliosis together with the occurrence of vimentin-positive astrocytes are also spatio-temporarily associated with ECM deposits [7,28].

In demyelinating CDV-induced lesions, aggrecan immunoreactivity decreased in the center of the lesions and accumulated in foamy macrophages. These findings were consistent with those of active MS plaques which showed an increased deposition of aggrecan in association with astrogliosis and within foamy macrophages at the lesion edges. In active plaque centers, there was a decrease in the aggrecan expression [39]. The brain-specific proteoglycan phosphacan was the only ECM molecule which was reduced in chronic demyelinating lesions. However, the role of phosphacan is controversially discussed. For example, several immunohistological and *in situ* hybridization studies have shown an up-regulation of phosphacan after brain injury [40–42]. In contrast, Western blot analyses and immunolabeling revealed a decreased expression of phosphacan in injured cerebral cortex and spinal cord in mice and rats [43–45]. In the human brain there is also constitutive expression of phosphacan [39]. The progressive reduction of phosphacan expression in this study is consistent with the majority of published studies [43–47]. Because proteoglycan molecules are able to inhibit neurite outgrowth *in vitro*, it was suggested that phosphacan may contribute to axonal regenerative failure after CNS injury in areas of reactive gliosis [48]. Due to those controversial findings, it is suggested that imbalances of the phosphacan expression in CDV-induced lesions may be responsible for unsuccessful axonal regeneration and/or remyelination in the distemper lesions [25].

Furthermore, in chronic CDV lesions, there was a dense deposition of vessel- and non-vessel-associated fibronectin. A similar response pattern was shown in the rat. Here, it was suggested that spinal fibronectin is elevated after peripheral nerve injury [49]. In active MS plaques, an extracellular fibronectin deposition was noted [50] which can be produced locally by endothelial cells and astrocytes as well as macrophages/microglia [51–54]. It was suggested that the failure of remyelination in chronic MS lesions could be due to the deposition of astrocyte-derived fibronectin [55]. Moreover, the deposition of fibronectin has previously been shown in TMEV-induced lesions [26]. It was also mentioned that fibronectin has a neuroprotective role in the traumatically injured mouse brain [56]. The effects of intralesional fibronectin deposition on reparative or regenerative processes indicate an important role of fibronectin in the pathogenesis of CNS inflammatory demyelinating events which occur in the course of distemper encephalitis.

Laminin immunoreactivity in the cerebella of controls and CDV-infected samples was largely restricted to the vascular basement membranes of gray and white matter and in the meninges. This finding is consistent with the physiological localization of laminin in normal adult CNS and in MS lesions, as described by several authors [52,57,58]. In contrast, in this study, the expression of laminin in chronic demyelinating lesions was nearly completely absent. Any reduction of the laminin expression in other lesion types was not observed. These results correlated with the observations of other authors who documented the absence of laminin in active MS lesions [50,58,59]. It was suggested that the absence of laminin in the parenchyma might be a major factor that prevents axonal regrowth [58]. It cannot be completely ruled out that the discrepancy in laminin expression in chronic lesions may alternatively be attributed to fixatives, staining techniques used and quality of the tissue. In fact, laminin immunoreactivity has previously been shown to depend on fixation time [60,61]. Furthermore, a decrease of laminin immunoreactivity by prolonged formalin fixation of up to 7 weeks was found [62]. Nevertheless, the failure to demonstrate a laminin up-regulation associated with astroglial scar formation suggests that other molecules may play a more significant role in preventing axon regeneration, as for instance shown in human spinal cord injury [63]. The failure to detect laminin in this study may also be due to the lack of laminin receptor expression of endothelial cells in case of an activation-induced matrix production [50,58,59].

The expression of type IV collagen in the cerebella of controls and CDV-infected dogs was detectable in vascular basement membranes as described in mouse, rat and man [20,26,58,64–66]. Additionally, type IV collagen immunoreactivity within demyelinating lesions was located in non-vessel-associated extracellular regions. In addition, at the edges of many demyelinated areas, type IV collagen immunoreactivity was clearly observed in the cytoplasm of macrophages. A similar response pattern was noticed by various authors in experimental spinal cord lesions in rats and cats [64–68]. In acute and chronic MS plaques, extracellular type IV collagen deposition was also shown [53]. Various studies demonstrated that the fibrous scar in the injured CNS is composed of a dense collagen IV meshwork, which acts as a binding matrix for other ECM components [68,69]. Excessive non-blood vessel-associated deposition of type IV collagen plays a pivotal role as a growth barrier for regenerating axons in the adult spinal cord [70]. Because of the additional non-blood vessel associated, net-like type IV collagen accumulation in demyelinating CDV lesions and the observed colocalisation with the other ECM components it can be assumed that in demyelinating distemper encephalitis the inhibitory effect on tissue regeneration is more prevalent. Similar to a TMEV study in mice, large amounts of type I collagen were detected within demyelinating CDV lesions [26]. In addition, an intralesional type I collagen deposition in experimentally injured spinal cord of cats was observed. As a source fibroblasts were suspected [67]. Furthermore, ingenuity pathway analysis (IPA) identified a network of a wide range of ECM components, like collagen type I, which was relevant for the development of chronic active plaques in MS [71]. Thus an inhibitory effect of the accumulated type I collagen in demyelinating distemper encephalitis lesions can also be suspected in the present study.

No expression of the CNS-specific proteoglycan brevican was detected in the white matter of the distemper cerebella. In contrast, it was demonstrated that brevican was considered to be a major constituent of the extracellular matrix of the human adult brain [72]. In TMEV infection, immunolabeling of brevican in murine spinal cord did not change with respect to distribution or intensity [26]. In contrast to those findings, the results of this study indicate that this proteoglycan is unlikely to contribute to the chronic demyelinating lesions in the cerebella of CDV-infected dogs. Furthermore, expression of decorin in the white matter of the investigated cerebella of controls and CDV-infected dogs was also not detected. In contrast to the findings of this study, in MS lesions there was an up-regulation of decorin in the perivascular space [73]. Also an increase in astrocytic decorin synthesis with subsequent extracellular accumulation in experimental traumatic CNS lesions in the rat has been described [74]. It was also shown that in TMEV infection none of the placebo animals expressed decorin-positive signals. Furthermore, in the same study, expression of decorin was detected mainly in demyelinating areas starting at 56 days post infection [26].

Furthermore, no expression of neurocan was observed in the CNS in this study. In some cases, there was a restricted expression in spinal nerve roots and perineuronal structures but not in the investigated white matter lesions. It seems that the antibody used in this study did not detect all canine neurocan variants. In humans and rats, there is a minimal production of neurocan in the gray and white matter [39,45,75]. After spinal cord injury in rats, neurocan levels increased within days in the parenchyma and peaked at 2 weeks post injury [45]. The lack of consistency between the findings of the present study in dogs and the results in rats and other species may be based on differences in the physiology of neurocan expression among the different species.

In a MS study, an interaction between ECM and immune or glial cells by forming a perivascular fibrosis was suspected [73]. A similar situation can be assumed for the CDV-infected animals because especially in chronic lesions, a massive perivascular infiltration of lymphocytes and macrophages is noted.

In general, intralesional activated astrocytes may represent the major source of different ECM molecules in CNS injury, resulting in glial scar formation [39,45,48,66,68,75,76]. In CDV-DL, the number of glial fibrillary acidic protein (GFAP)-positive astrocytes decreased in chronic plaques whereas the number of vimentin-positive, immature astrocytes increased compared to controls [7]. In contrast, in TMEV-infected mice, the number of GFAP-positive astrocytes and the amount of their processes continuously increased [26]. In conclusion, another source of ECM synthesis like vascular structures can be assumed in CDV-DL. Double labeling demonstrated that intralesional deposition of azan-positive material occurred to some extent with spatial association to von Willebrand factor-positive vascular structures in chronic distemper lesions, but was also found diffusely distributed in the lesioned area without direct vascular association. Additionally, factor VII immunohistochemistry showed a mild increase of vascular density in subacute and chronic lesions compared to NAWM and early lesions. Thus, the production of ECM components by vascular structures like endothelial cells can be assumed. An additional contribution of invading meningeal fibroblasts, as demonstrated in spinal cord injury models, is considered unlikely in CDV-DL lesions, because there is no primary meningeal tissue destruction, like in traumatic brain or spinal cord injury, and thus no considerable trigger for fibroblast proliferation and invasion into brain tissue [68,75,76].

As a pathogenetic mechanism for deposition and degradation of ECM molecules and thus progression of distemper lesions an imbalance of MMPs and their inhibitors TIMPs was suggested [77,78]. MMPs and TIMPs were prominently up-regulated in acute and subacute non-inflammatory distemper lesions. It was shown that they were mainly expressed by astrocytes and macrophages/microglia and by perivascular inflammatory cells in advanced lesions [78]. Accordingly, RT-qPCR analysis of the present study revealed an up-regulation of *MMP9*, *MMP12*, and *TIMP1* in CDV-infected dogs. However, *MMP13* and *MMP14* did not show a significant variation between control and diseased dogs.

In microarray analysis, *TIMP1* was the gene with the highest transcriptional up-regulation. In accordance to the results of RT-qPCR, mean expression values increased from acutely to subacutely infected animals, however microarray analysis showed subsequent increase of expression values for this gene in chronic lesions, whereas RT-qPCR detected a decrease of TIMP1-expression from subacute to chronic animals. TIMP1 is able to stimulate growth and inhibit apoptosis via activating phospho-inositol-3- kinase and MAPK signaling [79]. However, the precise function of TIMP1 in demyelinating diseases is not known in detail, yet an impairment of lesion progression and myelin loss was suggested [2]. Constitutive expression of TIMP1 in the CNS of transgenic mice, starting prenatally and persisting in high levels throughout the experiment, demonstrated a dose-dependent reduction of the clinical course and morphological lesions in EAE [80]. The authors suggested an inhibition of lymphocytic infiltration via astrocytic TIMP1 expression.

However, no significant differential expression was found for various MMP transcripts in microarray analysis. Nonetheless, expression values for *MMP2*, and *MMP9* had a similar trend in RT-qPCR and microarray analysis. Determining the correlation between microarray analysis and RT-qPCR of MMPs and their inhibitors, poor correlation was observed for *MMP13*, *MMP14*, and *RECK* in contrast to the high correlation of *TIMP1*. Different levels of sensitivity between the two methods most likely explain the discrepancy. For the Affymetix platform BioB is used as a hybridization control and is added to the hybridization cocktail with a concentration of about the sensitivity of the assay. Only about 15% of the 410 literature-based genes involved in synthesis and degradation of ECM exhibited expression values above the mean BioB expression. The low abundance of the respective genes was also reflected in the qPCR results. Moreover, correlation was shown to increase with the degree of change [81,82]. Below a fold change value of 1.4 to 1.6, generally no correlation is found between expression values obtained by microarray and RT-qPCR [81–83]. In our data, a fold expression greater than this cut off value was detected only for *TIMP1*, *MMP2* and *RECK*.

Besides *TIMP1*, microarray analysis pointed out a high increase in *CCL2* expression with highest values found in subacute CDV-infection. MMPs function as a regulator of several chemokines. CCL2 is a potent chemotaxin and plays a major role in the immune response by activation of monocytes, T-cells, NK-cells and basophils and is suggested to be responsible for the continued recruitment of inflammatory cells during chronic demyelinating diseases [84–86]. *CCL2* up-regulation highly contributes to the recruitment of peripheral macrophages and T-cells [87]. *Clc2-*deficient mice are resistant to autoimmune encephalomyelitis [88]. Despite the immunomodulatory function, CCL2 is suggested to directly influence remyelination by increasing the motility of oligodendrocyte precursor cells [89]. The production of CCL2 was suggested to be regulated by fibrillary collagens [73].

*FGFR2* exhibits significantly lower levels in acute and subacute lesions. An up-regulation of FGFR-2 was described in initial stages of remyelination in mice [90]. Further studies indicated, that FGF-2 inhibits oligodendrocyte precursor cell differentiation during development and remyelination [91]. However, it was shown recently that in mice deficient of *Fgfr1* and *Fgfr2* fewer differentiated oligodendrocytes and less remyelination are detectable, suggesting that FGF-signaling facilitates regenerative processes in chronic demyelination [92]. The opposing results emphasize the uncertainty about the influence of FGF-signaling during demyelinating disease and further studies are needed to elucidate these results.

Similar to a previous study upon TMEV infection, ECM protein accumulation and expression of remodelling-associated genes diverged to some extent [26]. As proposed in the earlier study, different observation periods in both methodological approaches may may have contributed to this discrepancy [26]. While immunohistochemistry is supposed to reflect an entire and overall view of altered protein production, microarray analysis is rather characterized by a snapshot-like picture of a particular transcriptional state [26]. Interestingly, the deposition and accumulation of ECM molecules in CDV-DL showed a similar temporal distribution as in TMEV-infected mice [26]. Most of the investigated ECM molecules in the spinal cord of experimentally TMEV-infected mice showed significant differences compared to mock-infected animal at 98 and 196 days post infection[26]. Considering data achieved from experimental CDV infection and lesion development in dogs, the accumulation of ECM molecules like type I and IV collagen and fibronectin as well as the downregulation of phosphacan were most prominent in subacute and chronic lesions which occur as early as 24–32 days post infection (subacute non-inflammatory) and after a minimum of 29–63 days post infection (inflammatory subacute and chronic lesions) [93–98]. These findings are also indicative of an earlier and more prominent activation of ECM synthesis in dogs compared to mice.

In summary, it could be shown that an increased expression of different ECM molecules occurred in CDV-DL. It is suspected that virally or immunopathologically induced insults lead to an activation mainly of glial cells (especially astrocytes), endothelial cells and inflammatory cells. Thus, an altered MMP/TIMP balance may be responsible for an intralesional accumulation of different ECM molecules and a downregulation of phosphacan expression. This environment especially in advanced distemper lesions might have inhibitory effects on regenerative processes like axonal sprouting and remyelination.

Further *in vivo* and *in vitro* studies are needed to elucidate the complex process of matrix production and degradation as well as its impact on regenerative attempts of the central nervous system in demyelinating diseases.

## Supporting Information

S1 Fig Histochemical stains in non-infected canine brains (controls, group 1). A: Cerebellum, white matter, control with bluish positive vascular walls. Azan stain. B: Cerebellum, white matter, control, red signal of vascular wall. Modified picrosirius red stain. C: Cerebellum, white matter, control, black signal around blood vessels. Gomori`s silver stain. All scale bars = 50 μm.
(TIF)

S2 Fig Immunohistochemical detection of aggrecan, phosphacan, fibronectin, collagen type I, collagen type IV and laminin in non-infected canine brains (controls, group 1). A: Cerebellum, white matter, control, minimal extracellular expression of aggrecan. B: Cerebellum, white matter, control, prominent phosphacan deposition. C: Cerebellum, white matter, control, mild expression of fibronectin in vascular walls (arrow) and in the cytoplasm of glial cells. D: Cerebellum, white matter, control, mild to moderate type I collagen expression associated with basement membranes (arrow). E: Cerebellum, white matter, control, mild to moderate type IV collagen expression around basement membranes of vascular walls (arrow). F: Cerebellum, white matter, control, minimal laminin expression associated with basement membranes of blood vessels. Immunohistochemistry (DAB), all scale bars = 50 μm.
(TIF)

S3 Fig Immunohistochemical detection of factor VIII in control brain tissue and different CDV lesion types as well as factor VIII/azan double labeling. A: Cerebellum, white matter, control, factor VIII-positive signal in endothelial cells (arrow). B: Cerebellum, white matter, acute lesion, mild to moderate labeling of factor VIII in blood vessels (arrows). C: Cerebellum, white matter, subacute lesion with inflammation, mild to moderate expression of factor VIII in endothelial cells (arrows). D: Cerebellum, white matter, chronic lesion, factor VIII expression in capillary endothelial cells surrounded by azan-positive ECM deposits (arrows). Note extensive reticular, extracellular, intralesional deposition of azan-positive material (arrowhead). Immunohistochemistry (DAB), all scale bars = 50 μm.
(TIF)

S1 Table Literature-based, manually created gene list comprising 410 genes associated with synthesis and degradation of extracellular matrix or fibrosis present on Affymetrix GeneChip canine genome 2.0 (Affymetrix, Santa Clara, USA). Displayed are the log_2_ transformed, GC- Robust Multiarray Averaging (RMA) preprocessed expression data, the fold change of expression values, p-value and adjusted p-values computed employing the Linear Models for Microarray Data (LIMMA) algorithm with p-value adjustment for multiple testing according to the False Discovery Rate algorithm developed by Benjamini and Hochberg from a previously published microarray study assessible via the ArrayExpress database (accession number: E-MEXP-3917; http://www.ebi.ac.uk/arrayexpress)[8].
(XLSX)
