# Supplementary figures and images for: Accumulation of Extracellular Matrix in Advanced Lesions of Canine Distemper Demyelinating Encephalitis
Source: PLoS One. 2016 Jul 21;11(7):e0159752. doi: 10.1371/journal.pone.0159752 (PMC4956304; doi:10.1371/journal.pone.0159752)

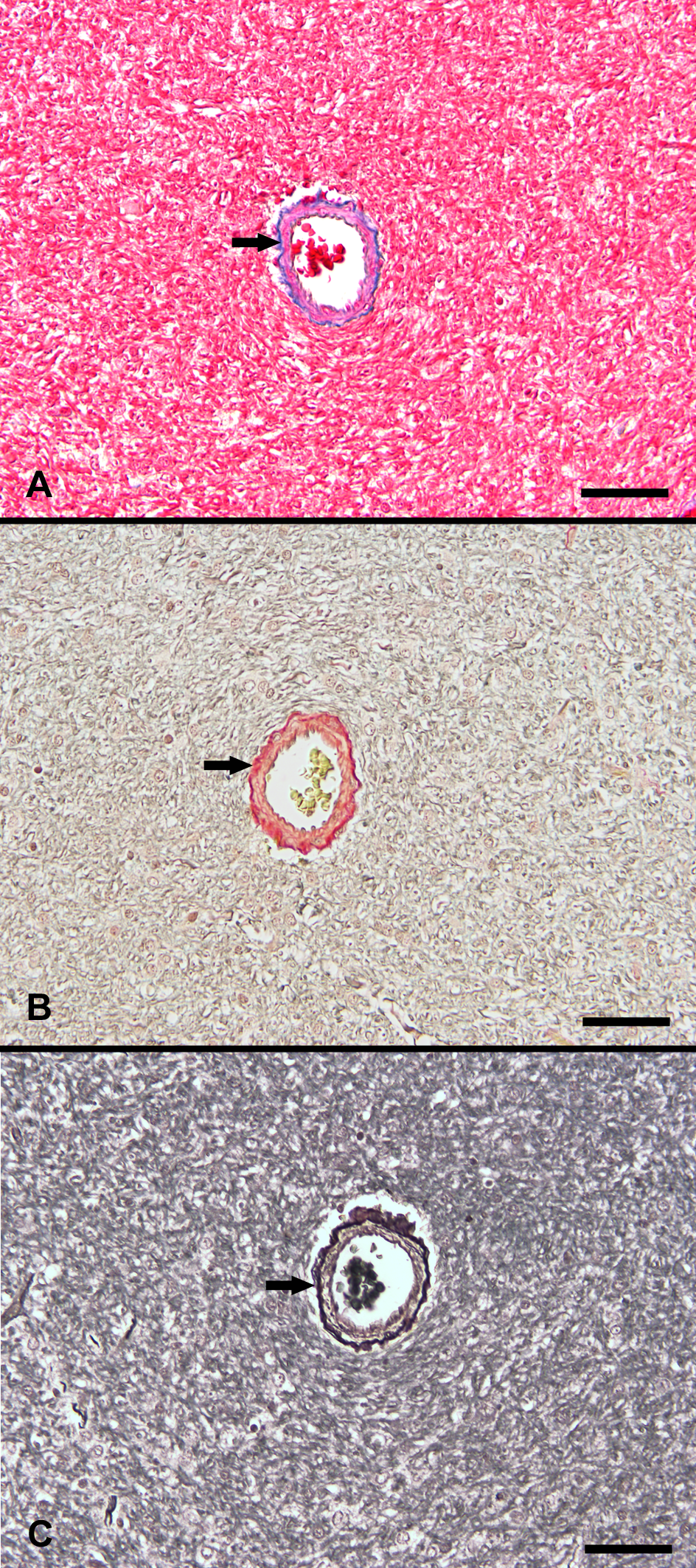

Supplement: S1 Fig — A: Cerebellum, white matter, control with bluish positive vascular walls. Azan stain. B: Cerebellum, white matter, control, red signal of vascular wall. Modified picrosirius red stain. C: Cerebellum, white matter, control, black signal around blood vessels. Gomori`s silver stain. All scale bars = 50 μm. (TIF) [file pone.0159752.s001.tif]

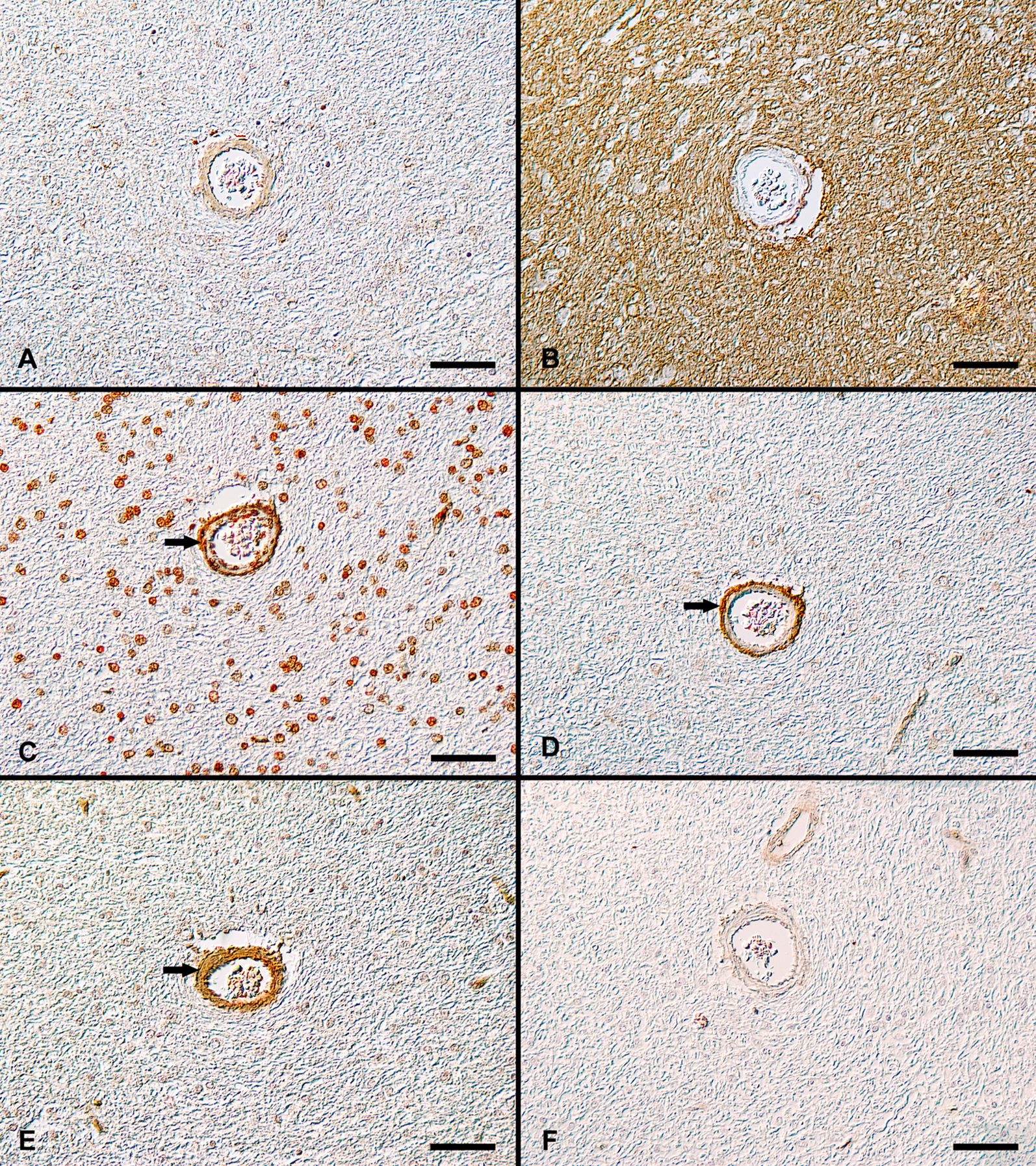

Supplement: S2 Fig — A: Cerebellum, white matter, control, minimal extracellular expression of aggrecan. B: Cerebellum, white matter, control, prominent phosphacan deposition. C: Cerebellum, white matter, control, mild expression of fibronectin in vascular walls (arrow) and in the cytoplasm of glial cells. D: Cerebellum, white matter, control, mild to moderate type I collagen expression associated with basement membranes (arrow). E: Cerebellum, white matter, control, mild to moderate type IV collagen expression around basement membranes of vascular walls (arrow). F: Cerebellum, white matter, control, minimal laminin expression associated with basement membranes of blood vessels. Immunohistochemistry (DAB), all scale bars = 50 μm. (TIF) [file pone.0159752.s002.tif]

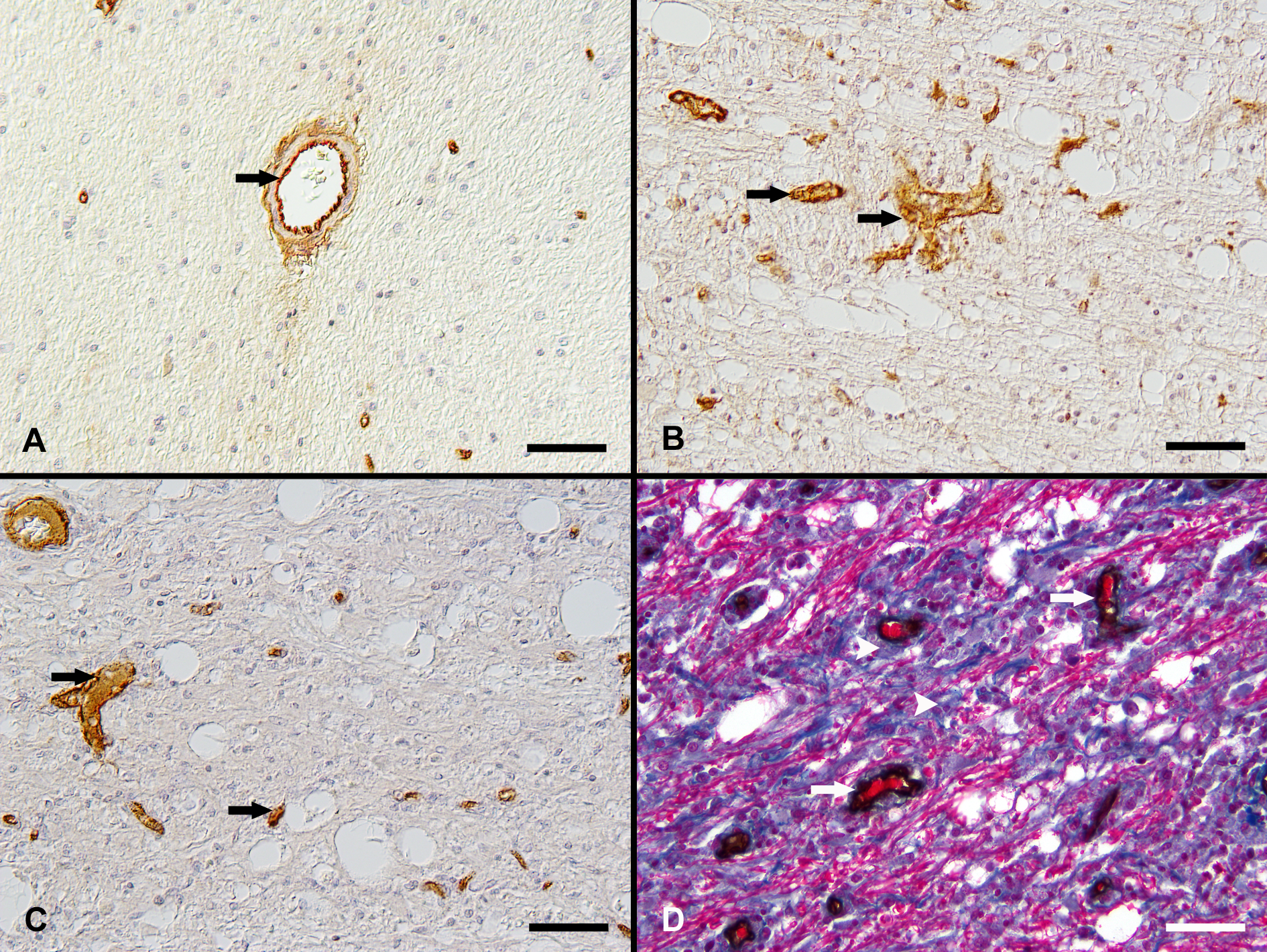

Supplement: S3 Fig — A: Cerebellum, white matter, control, factor VIII-positive signal in endothelial cells (arrow). B: Cerebellum, white matter, acute lesion, mild to moderate labeling of factor VIII in blood vessels (arrows). C: Cerebellum, white matter, subacute lesion with inflammation, mild to moderate expression of factor VIII in endothelial cells (arrows). D: Cerebellum, white matter, chronic lesion, factor VIII expression in capillary endothelial cells surrounded by azan-positive ECM deposits (arrows). Note extensive reticular, extracellular, intralesional deposition of azan-positive material (arrowhead). Immunohistochemistry (DAB), all scale bars = 50 μm. (TIF) [file pone.0159752.s003.tif]
